# Museums and art therapy: A bibliometric analysis of the potential of museum art therapy

**DOI:** 10.3389/fpsyg.2022.1041950

**Published:** 2022-11-22

**Authors:** Zhizi Wei, Chaofang Zhong

**Affiliations:** College of Teachers Education, Zhejiang Normal University, Jinhua, Zhejiang, China

**Keywords:** art therapy, museum education, healing, mental health, aesthetic intervention

## Abstract

In this paper, the current promotion of art therapy in museum development and the potential value of the combination of museum and art therapy on mental health are explored. Individuals who usually evade any treatment may experience art therapy in a museum setting as a release from their suppressed emotions. Furthermore, art therapy may enable them to discover how to express themselves, thereby reducing anxiety and promoting a sense of social belonging, which may be unattainable in other healing settings. Moreover, this literature review afforded us a preliminary understanding of issues in museum education and art therapy, which require further examination, including implications for China’s museum art therapy in practice and future research directions.

## Introduction

The significance of museums, which are an essential component of a lifelong learning system, has gained considerable recognition in recent years. In *Education 2030 Framework for Action* (Willockx and Dom, 2022), which was released by the United Nations Educational, Scientific and Cultural Organization, expands the mission of education to comprise an inclusive and equitable quality education and lifelong learning for all, affording everyone a fair opportunity. Museum education has an influence on the development of education. As an essential institution of social education, a museum’s education focus has shifted from spreading information about the protection and value of cultural relics to attracting the public to participate in meaningful experiences to achieve the purpose of healing and improving life (Kannangara, 2017). Visiting museums can inspire long-term self-reflection and cognitive outcomes, especially in relation to social awareness (Jackson et al., 2015). Anechini et al. found that museum environment experience plays an important role in children’s psychological recovery. In the museum learning and experience, they provided an assessment of the aesthetic attributes of the museum environment and benefits to children’s recovery (Mastandrea et al., 2021). It is a process of acculturation that relies on pedagogical methods, development, fulfillment, and the acquisition of new knowledge (Diaz, 2012).

Museum has gradually become a new medium to help human beings to connect with society and move toward “equality and inclusiveness.” Family therapists, art therapists, and social workers have all advocated for the effectiveness of museums in therapy. Van Lith (2016) spent many years employing museum resources to enhance relationships between individuals, families, and groups. Through the “Speak Objects” practice, McGhie (2008) created opportunities for troubled teenagers from various backgrounds to converse, think, and debate about museum objects, thereby assisting their self-actualization and self-exploration. In a case study, by assessing the impact of the Rovereto Museum of Contemporary Art on children’s learning and experience, it was demonstrated that children would get a sense of relaxation and happiness through aesthetic experience in museum education activities (Mastandrea et al., 2021). Research shows that museums provide a safe environment for the art therapy experience, thus promoting the process of cultural adaptation and providing health and experience with self-discovery.

During the COVID-19 pandemic, WHO has emphasized that new measures such as self-isolation have affected people’s normal activities, and it is necessary to prevent the increase of mental disorders and psychosocial problems, such as loneliness, anxiety, depression, and self-injury (Holmes et al., 2020). However, prevention to reduce the risk of mental disorders alone is not sufficient. It is imperative to determine various means to promote mental health (Keyes, 2007; Keyes et al., 2010). The guidelines for psychiatry and schizophrenia, which the National Institute for Health and Clinical Excellence in the United States published, indicate that art therapy can alleviate adverse symptoms such as psychosis (Attard and Larkin, 2016). The British Association of Art Therapy defined art therapy as “a form of psychotherapy that uses art media as its primary mode of expression and communication.” In such a context, art is not employed as a diagnostic tool but as a medium to address confusing and agonizing emotional problems (Wood et al., 2011). The focus of art therapy is its process, which tends to emphasize non-verbal communication and creative processes. In addition, art therapy can promote a safe environment of trust, thus empowering individuals to acknowledge and express strong emotions (Deshmukh et al., 2018).

**Figure A1:**
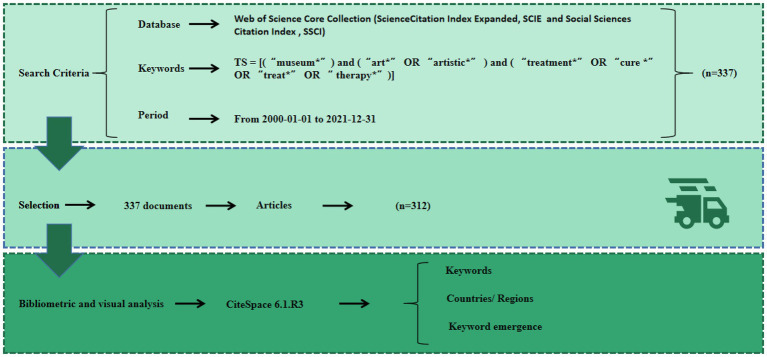
Search process overview. Document selection and flow chart for museum education and art therapy.

Since the 21 st century, many academic papers on museum education and art therapy have been published. However, these substantive scientific achievements are not conducive to our rapid grasp of insight into the future direction. In order to illustrate the importance of museum education and art therapy more fully, rigorous quantitative analysis and statistical analysis based on mathematical models are needed. Bibliometrics, citation analysis is a widely used new method of data-driven map literature (Chen et al., 2012), is widely used in research trend detection, agencies, national cooperation analysis, field changes (Carve et al., 2021), and may carry on the quantitative analysis model in the scientific literature, as well as to a research review and analysis of the emerging trends, It knows oriented quantitative function (Zhou et al., 2018; Tan et al., 2021). Early discussions on bibliometrics began in the 1950s (Wallin, 2005), suggesting that bibliometrics methods are not new. The use of bibliometrics is gradually expanding to all disciplines. Compared with the traditional review, the scientometric review is more systematic, comprehensive, and objective. In this paper, CiteSpace software is used to conduct a bibliometric analysis of 1,211 articles in the core database of the Web of Science and analyze the knowledge structure and trend direction of this field. The specific methods and retrieval strategies are shown in Figure A1. This database was chosen because it is more representative than the Scopus and Pubmed databases and reports articles in medicine, ecology, economics, and other fields.

## Methodology and data

### Data source and retrieval

To avoid bias due to daily database updates, the search was conducted on October 12, 2022, for articles published in the Web of Science Core Collection (Science Citation Index Expanded (SCIE), The Social Sciences Citation Index (SSCI), the search was conducted in the 21st century (January 1, 2000 to December 31, 2021), and articles published after January 1, 2022, were excluded because any collection from that year onwards would include incomplete bibliometric data from that year. The specific search parameters are: (TS = [(“museum*”) and (“art*” OR “artistic*”) and (“treatment*” OR “cure *” OR “treat*” OR “therapy* “). A total of 337 publications were obtained by precise search, retaining only research-based articles and excluding reviews and other articles, yielding 312 articles.

### Scientometric analysis

In this paper, the analysis tool of Web of Science (WOS) and CiteSpace software were used to make visual analysis and map drawing of museums and art therapy fields. CiteSpace, based on Java application program, is used for data analysis and visualization. It focuses on finding the key points, especially the turning points, in the development of a field, and converts the labor burden of some traditional content analysis into computer algorithms and interactive visualization, so as to facilitate a comprehensive analysis of the development of the field (Chen, 2006). The identified documents are extracted in WOS in plain text format and then imported into CiteSpace 6.1.R3.

## Outline of review

In this review, the theoretical origins of art therapy and the status of art therapy research in China are examined. A comprehensive assessment and comments on museum art therapy research results are provided. The purpose of this paper is to explore (a) the theoretical basis of art therapy for mental health recovery; (b) the potential value of museum art therapy; (c) the effect of museum art therapy on mental health.

## Literature review

### Theoretical origins of art therapy

Art therapy theory and psychoanalysis appear frequently in psychology theory. Gollnick’s (Gollnick, 1993) classical psychoanalytic theory revealed that art provides an “alternative satisfaction” of fantasy that individuals cannot attain in reality, thus reducing distress and initiating healing, Brownstein’s (Brownstein, 2017) analytical psychology verified the therapeutic effect of imagery creation. The new psychoanalytic school emphasized the integrating and adjusting effects of art therapy to the self. Moreover, while the humanism-oriented art therapists (Thomas, 2003) demonstrated the healing effect of creativity from educational practices, the transpersonal psychology school elaborated on the ultimate value of the human mind (spirit), its potential, and self-fulfillment. These provide the theoretical foundation of psychology for art therapy research (Table 1). Furthermore, research results have been applied to support the therapeutic properties of art (Franklin, 1999; Schure et al., 2008; Schimmels, 2020).

**Table 1:** The theoretical basis of art therapy research.

Theoretical basis	Research content	Research outcome
Freud’s classical psychoanalysis and art therapy	Freud merged art perspectives into his psychoanalytic theory, providing a valuable theoretical foundation for the therapeutic function of art	Freud revealed that the process of art creation is conducive to the expression of the subconscious and simultaneously noted that creation itself is a process of sublimation. He suggested that art provides an alternative satisfaction of fantasy that individuals cannot attain in reality, thus reducing distress and initiating healing
Jung’s analytical psychology and art therapy	Jung further developed Freud’s theory and developed analytical psychology. He proposed that personality comprises consciousness, the individual unconscious, and the collective unconscious. The goal of psychotherapy is to help individuals reveal the components of personality and subsequently integrate and perfect them to achieve self-realization	Jung proposed the therapeutic effect of imagery creation and the role of painting in the communication between consciousness and subconsciousness, supporting the healing function of art from a psychological perspective
New psychoanalysis and art therapy	The new psychoanalytic school essentially discusses personality structure and psychotherapy from aspects such as social environment, interpersonal relationships, and culture	The new psychoanalysis is not a tightly unified school. Its representatives demonstrate different theoretical preferences while also sharing common ground. First, the influence of social and cultural factors on individuals’ psychology and behavior is highlighted. Second, the vital role of family background and childhood experience (20)
Humanism and art therapy	Humanism advocates people-oriented practices. It regards the whole person (the uniqueness of each individual) as the research object, cares about human nature, values, and dignity, and studies healthy personality and self-realization	Humanistic-oriented art therapists reveal the healing effect of creativity. They have revealed that during the process of creating and sharing art, individuals can attain self-awareness, self-discovery, and self-integration so as to clarify the meaning of life and achieve self-realization (21)
Transpersonal psychology and art therapy	Transpersonal psychology emerged in the late 1960s and is currently flourishing. It mainly explores the ultimate value of the human mind (spirit), its potential, and self-fulfillment (22)	Transpersonal-oriented art therapy recognizes that imagery is healing and uses artistic expression as a way of exploring oneself and entering a non-ordinary (transcendent) state of consciousness

### The potential valuable role of museum art therapy

#### Museums as restorative environments

Attention restoration theory (ART; Kaplan and Kaplan, 1989) postulates that museums as restorative environments have the potential to promote mental health. Several studies have shown that there is a clear association between the characteristics of museums and the features of creating restorative experiences proposed by Kaplan (1995). Kaplan et al., presented four pieces of evidence that verify museums’ restoration properties. First, individuals display fascination because of extraordinary treasures in museums. Second, being away involves taking a break from ordinary daily life. Third, extent, both range, and coherence relates to the rich and coherent collections that are waiting to be explored. Finally, compatibility is related to how well the environment supports individuals’ visiting intentions and goals. Of the four, fascination, which is considered to be the core restorative characteristic necessary for a good environment, plays a vital role in ART (Kaplan, 1995). By providing a fascinating environment, museums attract the attention span of children effortlessly. This may facilitate their self-reflection. Studies have highlighted that museums provide adolescents with support for restoration as well as assistance that augments or optimizes adolescents’ mental health, which is clearly beneficial to children (Kaplan et al., 1993; Kelz et al., 2015; Laddis, 2019) analyzed the restorative qualities and benefits of art museums that are deemed as the essential public institutions of learning: learning and discovering, passive enjoyment, restoration, socialization, and self-actualization. Museum education can give people a sense of relaxation and make them positive again to deal with the stress and difficulties in life (Kaplan et al., 1993).

#### Research status of museum art therapy

**Figure 1:**
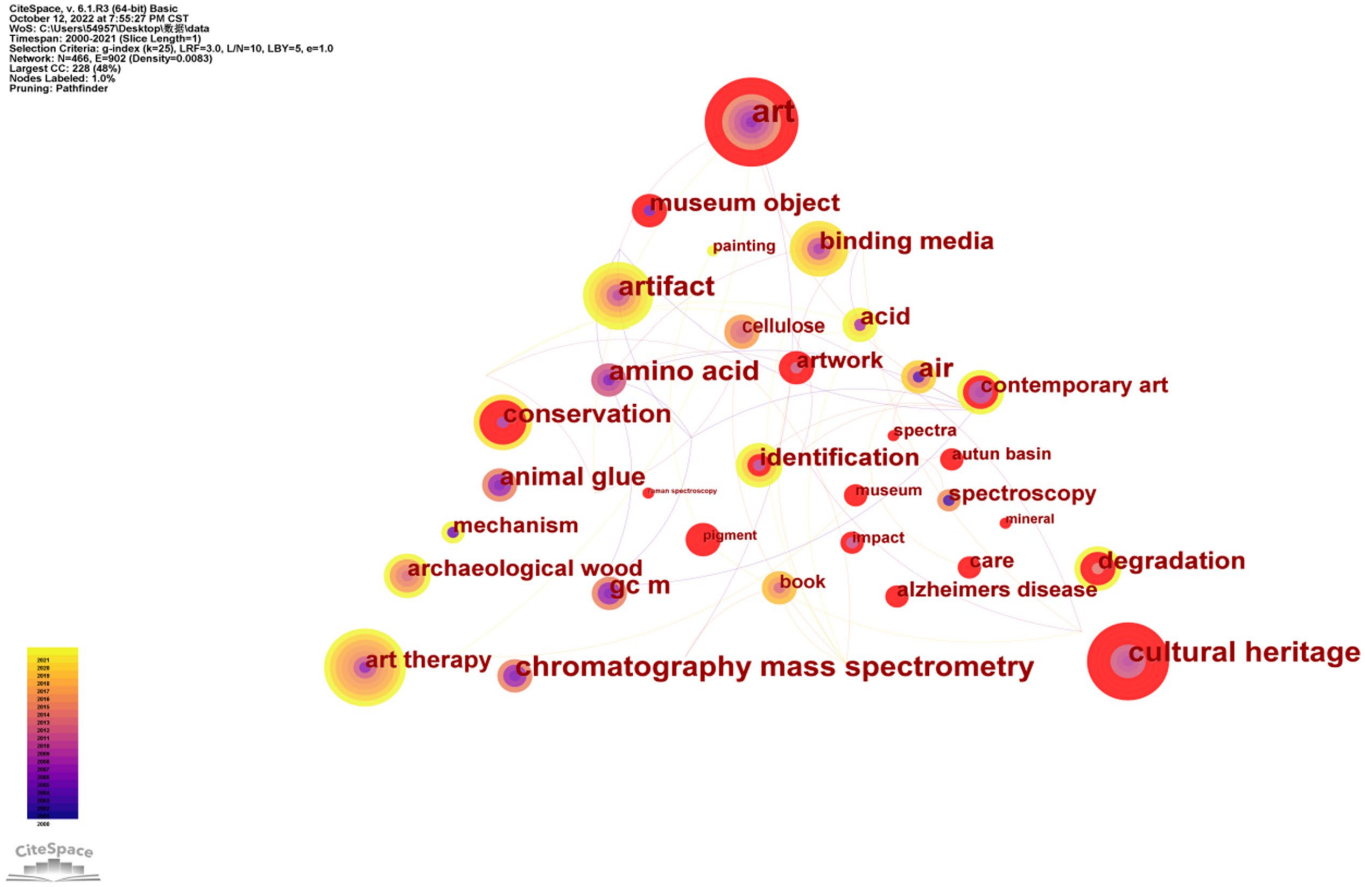
Co-occurrence analysis of keywords (*n* ≥ 3). (The nodes represent the categories of keywords. The size of nodes is proportional to the number of keywords. Links represent the connections between different keywords. The color of rings and links corresponds to the year. Purple rings indicate high centeredness.)

According to the bibliometrics theory, keywords represent research status and trends in a research field (Li et al., 2016). They reflect the focus of an article or an author (Liu et al., 2012). Based on Figure 1, the connection between nodes represents the interrelation between keywords, the color of the connection represents the time and year of its occurrence, the size of the node represents the occurrence frequency of a keyword, and the larger the node is, the higher the occurrence frequency of the keyword is, and vice versa. Thus, it can be seen that “art,” “museum object,” “art therapy,” and “cultural heritage” are popular research topics, indicating that art projects and museum exhibits in museum art therapy are a current area of research focus.

Burst keywords can test whether a research area is a research priority at a particular time (Yan et al., 2020) and explore emerging topics. Therefore, we used CiteSpace software to analyze the top 30 keywords with the strongest citation bursts (Table 2). Since 2016, when “contemporary art,” “care,” and “reminiscence” broke out, researchers started to attach the importance of the recovery effect of art to human mental health. The sustained burst of “cultural heritage” and “art” in 2021 shows that museum art therapy would become a focus in this field in future research. It can also be demonstrated from some examples that museum art therapy still has great potential. For example, Canada’s oldest museum, the Montreal Museum of Fine Arts (MMFA), has always been regarded as avant-garde. In such a surreal era, as the pandemic rampaged through humanity, many North American museums, including the MMFA, acted to help people overcome the confusion and darkness of global isolation, thus museum therapy has developed rapidly (Picard, 2001).

**Table 2:** Top 30 Keywords with the strongest citation bursts.

Keywords	Year	Strength	Begin	End	2000–2021
Medical history	2000	1.35	2001	2001	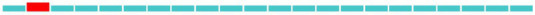
Rheumatoid arthriti	2000	1.3	2001	2002	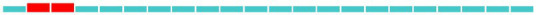
Phylogeny	2000	1.3	2011	2011	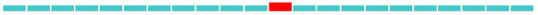
Museum object	2000	1.73	2012	2013	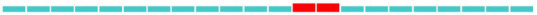
Radiation	2000	1.29	2012	2012	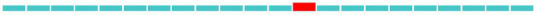
Conservation	2000	1.27	2012	2015	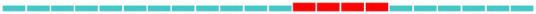
Raman spectroscopy	2000	1.91	2014	2014	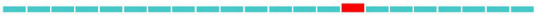
Identification	2000	1.62	2014	2014	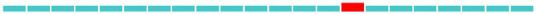
Resin	2000	1.27	2014	2014	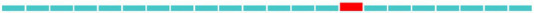
Media	2000	1.27	2014	2014	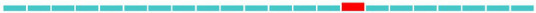
Spectra	2000	1.89	2015	2015	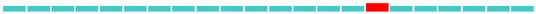
Mineral	2000	1.89	2015	2015	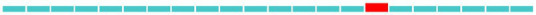
Autun basin	2000	1.71	2015	2016	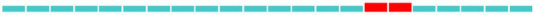
Elderly people	2000	1.26	2015	2015	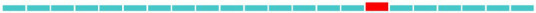
Dementia	2000	1.26	2015	2015	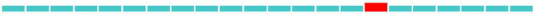
Contemporary art	2000	1.68	2016	2016	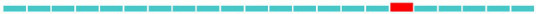
Degradation	2000	1.61	2016	2018	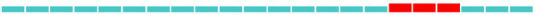
Care	2000	1.58	2016	2018	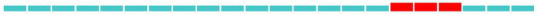
Reminiscence	2000	1.27	2016	2016	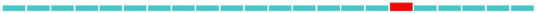
Atr ftir	2000	1.27	2016	2016	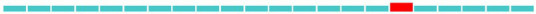
Iron corrosion	2000	1.27	2016	2016	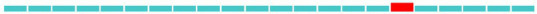
Cultural heritage	2000	2.51	2017	2021	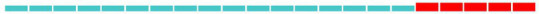
Museum	2000	2.27	2018	2019	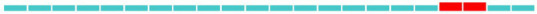
Artwork	2000	2.14	2018	2019	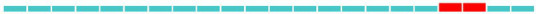
Impact	2000	1.85	2018	2018	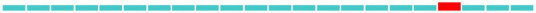
Alzheimers disease	2000	1.7	2018	2019	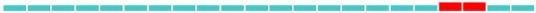
Caregiver	2000	1.27	2018	2018	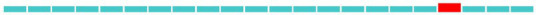
Intervention	2000	1.27	2018	2018	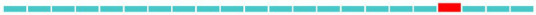
Art	2000	1.36	2019	2021	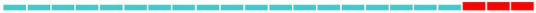
Pigment	2000	3.35	2020	2021	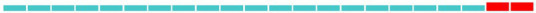

Each blue node in the table represents 1 year, a total of 22 blue nodes (22 years), where the red nodes represent the year in which the keyword outbreak occurred.

**Figure 2:**
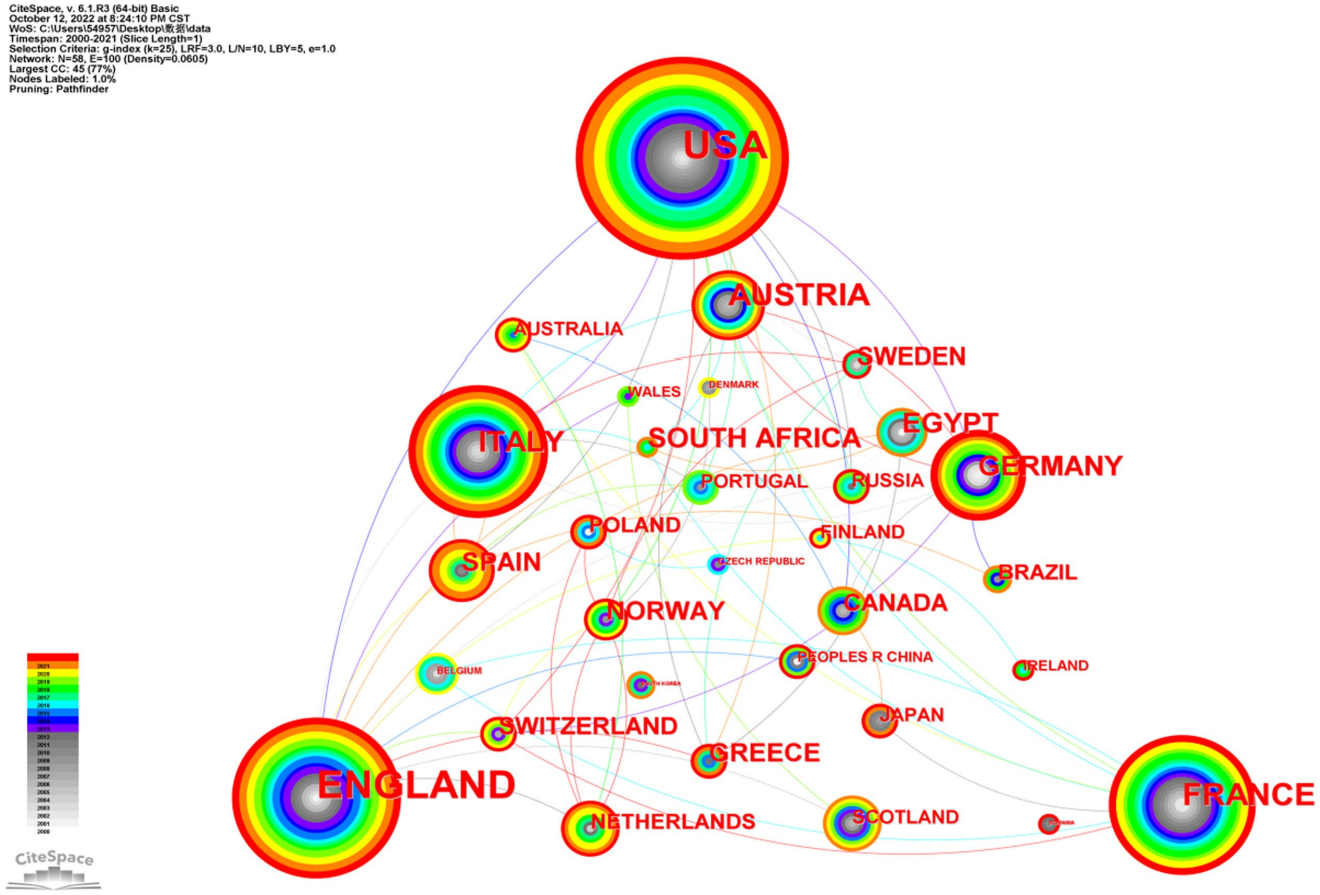
Country cooperation knowledge graph (*n* ≥ 3). (Nodes represent the category of countries. The size of nodes is proportional to the number of documents in the country. Links represent cooperative co-occurrence relationships between different countries. The color of rings and links corresponds to the year. Purple rings indicate high centeredness.)

By looking at the size of the nodes and the thickness of the lines, the contributions of individual countries and their relationships can be determined (Figure 2). The larger the circle, the more publications the country has in that field. Research shows that the United States occupies a central position in this field, along with the United Kingdom and France. In this area, the United States often cooperates with the United Kingdom, France, Germany, and Italy, and the United Kingdom has extensive cooperation with Switzerland, the Netherlands, Italy, and China.

#### The role of museum art therapy on mental health

The ability to offer meaningful experiences for individuals of different backgrounds may be regarded as museums’ social value. Marxen (2009) referred to the museum environment as a “potential space” for art therapy, fostering creative expression in “a safe empathic atmosphere” (p. 133). Among others, Hamil, Alter-Muri, McNiff, Marxen, and Salom (Trent, 1992; Fulcheri, 2002; Marxen, 2009; Salom, 2011; Ali and Haen, 2019) considered museums as protective places for healing and transformative experiences. These experiences include supporting a cohesive sense of self (identity), a sense of universality (belonging), and the meaning of relationships (validation) (Fulcheri, 2002). Art therapy has been shown to possess enhanced healing potential in promoting mental health through partnerships with museums and galleries (March et al., 2004). Museums address health issues by providing “non-traditional instructional services, gaining a vital role in community connection,” (Ioannides, 2016; Thaler et al., 2017) which has been confirmed by the art psychotherapy program at EMST (National Museum of Contemporary Art Athens). Research has revealed that museum collections increase participants’ self-esteem, confidence, and creativity. They also validate their experiences, promote critical thinking, increase open-mindedness, and stimulate intelligence (Linesch, 2004; Beel, 2009; Leonard, 2010; Bennington et al., 2016). From a psychological perspective, it is evident that the museum environment is a crucial part of the *perceived experience*. Every experience is part of an individuals’ interaction with their human and physical environments (Penfold, 2017). Museum inspires children’s feelings and emotions when activities and experiences related to art therapy are organized, allowing their minds to be receptive to new ideas. Tröndle and Tschacher (2012) found that artistic experiences in a museum are closely related to the visitors’ tour through the museum space. British Alain De Botton and John Armstrong held the *Art Is Therapy* exhibition, which aimed to present a series of emotional themes vividly that are conducive to restoring mental balance. Alain De Botton asserted that art therapy in museums can assist in finding solutions to numerous problems in life such as reducing forgetfulness, bringing hope, demonstrating dignified sorrow, expressing painful feelings, promoting mind connections, helping one achieve mental balance, and guiding one in self-awareness (Serretti et al., 2004). The *See You at the Museum* project, which was presented by The Museum of Modern Art of New York, focused on guided tours and interactions. It was a healing exhibition for patients and their families. Canadian Doctors prescribed a *museum prescription* (Salom, 2011) and announced the collaboration between the Association of French-speaking Physicians of Canada and the MMFA. Helena Boyle, vice president of the Association of French-speaking Physicians, related that visiting museums has been proven to increase the secretion of serotonin, which is a neuroregulatory hormone that makes one feel happy. It is commonly known as the *happy factor*.

Research has demonstrated that appreciating abstract modern painting in art galleries, that is, in an artistic context that requires a distant artistic perspective may evoke strong aesthetic emotions, improving individuals’ sense of well-being (Freedberg and Gallese, 2007; Leder et al., 2014; Consoli, 2019). Such experiences leave visitors with a sense of temporary separation from reality. Subsequently, they return to everyday life with a new awareness, specifically a feeling of being a part of “something bigger” (Kaplan et al., 1993). The combination of museum and art therapy, an innovative project, can help children connect with those who have similar feelings of loss, anger, and sadness so that they can regain their sense of security and control (Rochford, 2017). In accordance with the noted testimonies and based on restorative principles, it can be concluded that museum art therapy could possess realistic therapeutic functions. At the same time, the function of art therapy is strengthened. The value of this method includes alleviating the resistance of the community to treatment (Salom, 2011).

## Discussion

In this paper, the current promotion of art therapy in museum development and the potential value of the combination of museum and art therapy on mental health are explored. Individuals who usually evade any treatment may experience art therapy in a museum setting as a release from their suppressed emotions. Furthermore, art therapy may enable them to discover how to express themselves, thereby reducing anxiety and promoting a sense of social belonging, which may be unattainable in other healing settings. Moreover, this literature review afforded us a preliminary understanding of issues in museum education and art therapy, which require further examination, including implications for China’s museum art therapy in practice and future research directions.

## Future research directions

The museum’s distinct multi-media environment and various artifacts and art pieces are the most optimal place to conduct treatments. The education form of museums is linked to art therapies, which have demonstrated considerable potential in enhancing the treatment of psychological trauma during the COVID-19 pandemic and becoming an aesthetic intervention that can encourage positive changes in physiology and emotions.

The strength of museums can be found in creating new characters, stimulating new conversations, and inspiring new hopes. Currently, museums’ roles are increasingly diverse, prominently as mediators of individuals’ health and well-being and engines for social change (Betts, 2011). Some studies have shown that art is good for the soul and can improve both physical and mental well-being. Researchers in Canada investigated whether these art-based benefits could be delivered digitally through virtual museum tours (Beauchet et al., 2022). Amid the contemporary media background and in the process of intermingling with art therapy, the significance of the existence of museums and their unique educational function concerning things and benefits to people are recognized. Museums create significant interactions that encourage, support, and employ the relationship between people and things in new ways. This unique ability opens up imaginative and realistic frontiers for the healing potential of museums.

The relationship between museum education and art therapy is confirmed in this study. Furthermore, museum education’s potential functions and specific practices in art therapy for treating mental health are highlighted. The findings encourage future research on the practical and beneficial psychological effects a museum visit could generate. Moreover, museum education should increase the use of augmented reality, virtual reality, and mixed reality technology, thus expanding the scope of museum art therapy beyond physical constraints and creating VR space for aesthetic experiences. Museum education should also take advantage of the online museum education community, allowing people in different countries and regions to enjoy artwork online, express different opinions freely, and listen to each other.

## Limitations and conclusion

In this paper, a new bibliometric method was adopted to clarify the development and trends of this research to fill the gap in the research of museum art therapy. Thus, this field’s main progress and new insights were identified more effectively. The results show that researchers are more and more interested in the research of museum art therapy. Through the combination of keyword co-occurrence, keyword burst analysis, and national co-occurrence network analysis, possible research directions in the future are proposed. In the future, the field of museum art therapy will gradually move towards more diversified cross-country cooperation, and more museum art therapy projects will be developed through scholars’ cooperation and local cultural heritage characteristics. Therefore, further research is needed to solve the specific implementation mode of museum art therapy to demonstrate the participation mode and recognition of museums in art therapy.

This study is small-scale in nature. It is recommended that future research should explore the relationship between museum education modes in art therapy between patients with different clinical, physical, and mental states. Furthermore, follow-up studies are essential to assess the impact of museum art therapy on the short- and long-term effects of treatments in different treatment groups.

## Author contributions

ZW and CZ performed the conception and design of study. ZW wrote the manuscript. CZ contributed to the manuscript revision. All authors contributed to the article and approved the submitted version.

## Funding

This study was supported by Open Research Fund of College of Teacher Education, Zhejiang Normal University [grant number jykf22046].

## Conflict of interest

The research was conducted in the absence of any commercial or financial relationships that could be construed as a potential conflict of interest.

## Publisher’s note

All claims expressed in this article are solely those of the authors and do not necessarily represent those of their affiliated organizations, or those of the publisher, the editors and the reviewers. Any product that may be evaluated in this article, or claim that may be made by its manufacturer, is not guaranteed or endorsed by the publisher.
